# The Role of Redox-Regulating Enzymes in Inoperable Breast Cancers Treated with Neoadjuvant Chemotherapy

**DOI:** 10.1155/2017/2908039

**Published:** 2017-11-19

**Authors:** Nelli Roininen, Kirsi-Maria Haapasaari, Peeter Karihtala

**Affiliations:** ^1^Department of Oncology and Radiotherapy, Medical Research Center Oulu, Oulu University Hospital and University of Oulu, Oulu, Finland; ^2^Department of Pathology, Medical Research Center Oulu, Oulu University Hospital and University of Oulu, Oulu, Finland

## Abstract

Although validated predictive factors for breast cancer chemoresistance are scarce, there is emerging evidence that the induction of certain redox-regulating enzymes may contribute to a poor chemotherapy effect. We investigated the possible association between chemoresistance and cellular redox state regulation in patients undergoing neoadjuvant chemotherapy (NACT) for breast cancer. In total, 53 women with primarily inoperable or inflammatory breast cancer who were treated with NACT were included in the study. Pre-NACT core needle biopsies and postoperative tumor samples were immunohistochemically stained for nuclear factor erythroid 2-related factor 2 (Nrf2), Kelch-like ECH-associated protein 1 (Keap1), thioredoxin (Trx), and peroxiredoxin I (Prx I). The expression of all studied markers increased during NACT. Higher pre-NACT nuclear Prx I expression predicted smaller size of a resected tumor (*p* = 0.00052; *r* = −0.550), and higher pre-NACT cytoplasmic Prx I expression predicted a lower amount of evacuated nodal metastasis (*p* = 0.0024; *r* = −0.472). Pre-NACT nuclear Trx expression and pre-NACT nuclear Keap1 expression had only a minor prognostic significance as separate factors, but when they were combined, low expression for both antibodies before NACT predicted dismal disease-free survival (log-rank *p* = 0.0030). Our results suggest that redox-regulating enzymes may serve as potential prognostic factors in primarily inoperable breast cancer patients.

## 1. Introduction

Breast cancer is the most common cancer among women worldwide, and it is the fifth most common cause of death from cancer overall [1]. In Finland, 31% of the all invasive cancers in women were breast cancers causing 16% of all female cancer deaths during the years 2008–2012 (Association of the Nordic Cancer Registries). If the cancer is already widely locally advanced at the time of diagnosis and thus cannot be safely removed surgically, presurgical neoadjuvant chemotherapy (NACT) to shrink the primary tumor can be administered. The current Finnish breast cancer guidelines restrict NACT mainly to the patients with primarily inoperable disease due to inflammatory or widely locally advanced breast cancer. These cancers usually have an aggressive biological profile, and without efficient predictive factors, valuable time may be spent on ineffective chemotherapy. On the other hand, the breast cancer patients getting a complete response for NACT have usually an excellent outcome [2]. To save time and avoid ineffective chemotherapy regimens, more accurate prognostic factors for the chemotherapy responsiveness and aggressiveness of the breast cancer are needed.

Reactive oxygen species (ROS) are involved in cellular processes that favor cell migration and adhesion [3, 4]. Moderate oxidative stress accelerates carcinogenesis [5], while high ROS concentration leads to apoptosis and senescence [6]. Oxidative stress markers and antioxidant enzyme levels may therefore be useful predictive and prognostic biomarkers in cancer. Among the most potent antioxidant enzymes are peroxiredoxins (Prx) I–VI. Prxs are highly conserved through the living organisms from bacteria to human, indicating the importance of these enzymes [7, 8]. Although Prxs contribute to doxorubicin resistance of breast cancer cells *in vitro*, they have also breast cancer-suppressive properties via p53 and c-Myc inhibition [9, 10]. Similarly, human thioredoxin (Trx, cytoplasmic and nuclear) is involved in many physiological redox reactions but is also associated with increased hypoxia-induced factor 1*α* and vascular endothelial growth factor production and also chemoresistance *in vitro* [11–13].

Nrf2 (nuclear factor erythroid 2-related factor 2) belongs to the cap'n'collar (CNC) bZIP transcription factors [14]. Under normoxia, Nrf2 is constitutively driven to proteosomal degradation by its proximal regulator, Keap1 (Kelch-like ECH-associated protein 1). When the cell is exposed to oxidative stress, Nrf2-Keap1 interaction is disturbed and Nrf2 relocates from the cytoplasm to the nucleus where it complexes with small maf proteins and upregulates genes with an antioxidant response element (ARE) in their regulatory regions. Nrf2 has been suggested to play an essential role in the development of chemoresistance [15]. Therefore, Prx enzymes, Nrf2 transcription factor, Trx, and Keap1 are all markers that should be studied for predictive and prognostic use in malignancies.

Most chemotherapy agents act directly or indirectly on the excessive production of ROS [8, 16]. Anthracyclines (such as epirubicin and doxorubicin) and taxanes (usually docetaxel and paclitaxel) are the standard of care in breast cancer chemotherapy, including the neoadjuvant setting. Anthracyclines bind to metals, such as iron, and form drug-metal complexes. This kind of a complex is capable of producing iron-mediated oxidative stress reaction in the cell that leads to covalent modification of guanine bases of DNA [17]. The main mechanism of action of taxanes is based on the disruption of microtubule function, but they also exert at least their adverse effects via increased ROS production [18, 19].

The aim of the current study was to evaluate the possible association between major redox-regulating proteins and chemoresistance in a cohort of patients with primarily inoperable breast cancers treated with NACT. The prognostic value of the studied proteins was also assessed, both in pre-NACT and postoperative samples. As the main finding, immunohistochemical pre-NACT Keap1 and Trx expressions appear to predict especially poor outcome in these patients.

## 2. Materials and Methods

### 2.1. Patients and Samples

The study included 53 breast cancer patients, who at the time of diagnosis were inoperable due to local invasion or inflammatory breast cancer. The patients were treated with NACT in Oulu University Hospital, Finland, during the years 2000–2015. All the patients received a minimum of two NACT cycles (ranging from 2 to 16 cycles, median 6 cycles) (Table 1). All patients underwent mastectomy and axillary evacuation with radical intention after NACT. The response for NACT was classified as a complete response (no viable cancer cells in breast or lymph nodes after surgery), a partial response, a stable disease (no radiological change in tumor size during the NACT), or a progressive disease. The mean age at diagnosis was 56.4 (32–77), and the mean follow-up time was 43.8 months (3–112). None of the patients had earlier diagnosis of invasive breast cancer. Tumor properties and patient data were collected from medical records and are presented in Tables 2 and 3.

A core needle biopsy sample before NACT and a resected tumor sample after NACT were obtained from each patient in the study. Pre-NACT tumor sizes were available from magnetic resonance imaging in 38 (71.7%) patients and from ultrasound in 13 (24.5%) patients and were unmeasurable in 2 (3.8%; tumor filled the whole breast) patients. The mean tumor size based on imaging at the time of diagnosis was 54.6 mm (10–140 mm), and after NACT, it was 31.2 mm (0–90 mm). The average postoperative tumor size measured from the resected tumor sample was 39.1 mm (0–150 mm).

Patients were classed after the TNM classification, and the histopathology was evaluated according to the current WHO classification [20]. Estrogen receptor (ER), progesterone receptor (PR), and Ki-67 expressions were analyzed by immunohistochemistry as described previously [7]. HER2 expression was determined by immunohistochemistry, and when an HER2-positive result appeared, gene amplification status was determined using chromogenic in situ hybridization. Cancers with six or more gene copies were considered *HER2* positive [21].

### 2.2. Immunohistochemistry

Staining was performed following the routine protocol in the Department of Pathology, Oulu University Hospital. Tissue sections (4 *μ*m) were cut from the paraffin-embedded blocks. After deparaffinization in xylene and rehydration in graded alcohol solutions, the sections were heated in a microwave oven for 2 min (800 W) + 10 min (150 W) in citrate buffer (pH 6.0) and incubated in room temperature for 20 min. Immunostaining for Nrf2 was done the same, but the heating time at 150 W was 15 min in Tris-EDTA buffer (pH 9.0). The sections were then rinsed in distilled water and phosphate-buffered saline with TWEEN (PBS-TWEEN), incubated in endogenous peroxidase-neutralizing solution (Dako S2023, Dako A/S, Glostrup, Denmark) for 5 min, and washed twice for 5 min in PBS-TWEEN. The Keap1 and Prx I sections were incubated in protein block solution for 5 min.

The preprocessed slides were then incubated for 1 hr at room temperature with the monoclonal anti-Nrf2 (ab62352, EnVision detection system, Dako A/S, Glostrup, Denmark), anti-Keap1 (ab66620, Novolink Polymer Detection System, Leica Biosystems Newcastle Ltd., Newcastle, UK), anti-Trx (#2429, Cell Signaling Technology, Dako A/S, Glostrup, Denmark), and anti-Prx I (LF-PA0095, Novolink Polymer Detection System, Leica Biosystems Newcastle Ltd., Newcastle, UK) antibodies (dilutions 1 : 400, 1 : 800, 1 : 600, and 1 : 150, resp.). The Keap1 and Prx I slides were then incubated for 30 min in antibody-blocking solution. All the slides were finally incubated with a biotinylated secondary antibody and avidin-biotin-peroxidase complex (Novolink polymer for Prx I and Keap1, Envision polymer K5007 for Trx and Nrf2). Two rinses (5 min each) were performed with PBS following each step of the immunostaining procedure. The color was developed with incubation of 3 min with diaminobenzidine tetrahydrochloride (K5007, EnVision detection system, Dako A/S, Glostrup, Denmark). The slides were rinsed in distilled water, counterstained with Mayer's hematoxylin, washed, dehydrated, cleared, and mounted with Depex (BDH, Poole, UK). In negative controls, the primary antibody was omitted. Samples unrelated to the study material that were known to react with the various indicator antibodies were used as positive controls.

Two of the authors (NR and KMH) performed the evaluation of immunostaining for the tumor cell cytoplasm and nuclei. The intensity of the staining of both cell compartments was evaluated as 0 (negative), 1 (weakly positive), 2 (moderately positive), or 3 (strongly positive). The amount of stained cells was reported as percentages (0–100) out of all malignant cells. A histological sum score, H score, was computed by multiplying the intensity and staining percentage scores resulting in scale of 0–300 [22]. The H score allows areas with different intensities to be taken into account. Separate H scores were created for both nuclear and cytoplasmic immunostaining. Raw H scores were used in statistical analyses, with the exception of Kaplan-Meier analysis, where two-classed variable was created based on the receiver operating characteristic (ROC) analysis of the H score.

### 2.3. Statistical Analyses

For statistical analyses, ER, PR, Ki-67, and HER2 expressions and grade were recorded as mentioned in the postoperative pathoanatomical diagnosis (PAD). If unavailable in postoperative PAD, the assessments from the preoperative core needle biopsy were used. ER and PR expressions were classified into either negative (<1% of positivity) or positive (1–100% of tumor cells positive). Ki-67 was divided into either negative to moderate (0–30%) or high (>30%). Grade was divided into either I-II or III for statistical analyses. Tumor size was processed as millimeters, and the number of nodal metastases was also treated as a continuous variable.

IBM SPSS Statistics version 22.0.0.0 for Mac (IBM Corporation, Armonk, NY, USA) was used for statistical analysis. Two-classed variables (ER, PR, Ki-67 grade; the presence of multifocal disease; and bilateral breast cancer) were tested against H scores with the independent samples Mann-Whitney *U* test. Continuous variables were correlated with two-tailed Spearman's test, with the correlation coefficient. The Wilcoxon test was applied when comparing core needle biopsy H scores to postoperative H scores. Survival was analyzed by using the Kaplan-Meier method with the log-rank and Breslow tests. The endpoint in breast cancer-specific survival (BCSS) was the confirmed death due to metastatic breast cancer while in disease-free survival (DFS), the endpoint was either local relapse or distant metastasis, whichever occurred earlier. Reliable multivariate analysis could not be performed due to a low number of samples. *p* values < 0.05 were considered significant.

### 2.4. Ethical Considerations

This study was approved by the Local Ethics Committee of the Ostrobothnia Hospital District (114/2011, amendment 23.2.2015) and the National Supervisory Authority for Welfare and Health (1339/05.01.00.06/2009).

## 3. Results

**Figure 1:**
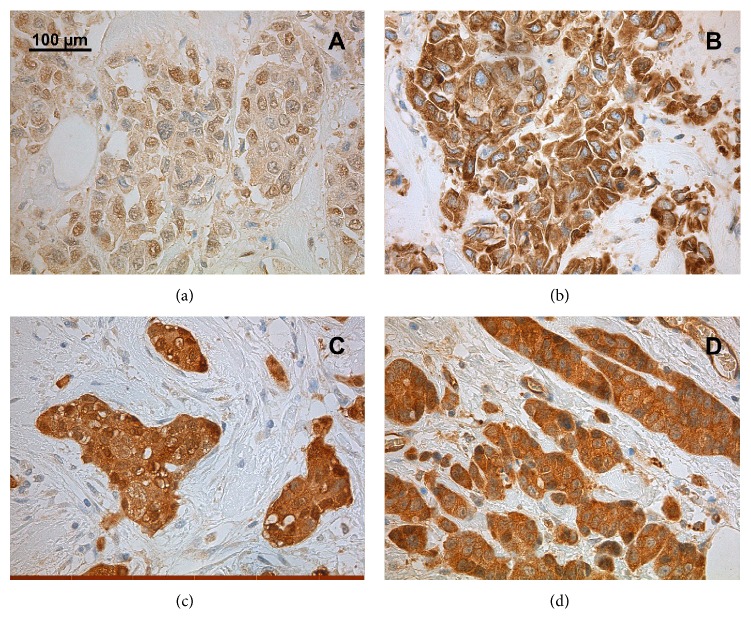
Immunohistochemical detection of protein levels of Keap1 (a), Nrf2 (b), Trx (c), and Prx I (d) in postoperative samples of NACT-treated patients. The figures represent samples with weakly positive (a), moderately/strongly positive (b), and strongly positive (c, d) cytoplasmic staining. None of the tumor samples were totally negative, but negative staining can be seen in connective tissue (c, d). Nuclear staining is negative in figures (b) and (d) and partly moderately positive in (a) and (c).

In pretreatment samples, some positive cytoplasmic immunostaining for all antibodies was detected in almost all the samples (Figure 1). Due to exhaustion of blocks or occurrence of nonrepresentative areas, especially in core needle biopsies, the immunostaining for some patients could not be reliably evaluated. Keap1 and Prx I showed at least some cytoplasmic expression in all pretreatment samples, and for Nrf2 and Trx, 94.6% of the samples showed at least some positivity (Table 4). Preoperative nuclear expression was detected in 45.3%, 45.9%, and 64.9% of the samples for Keap1, Prx I, and Trx, respectively (Table 4). Nrf2 was negative for nuclear staining in all samples.

Both the cytoplasmic (*p* = 0.0015) and nuclear (*p* = 0.0013) expressions of Trx were increased during the NACT. High cytoplasmic Trx expression in postoperative samples was associated with negative ER expression (*p* = 0.027). Elevated pretreatment cytoplasmic Nrf2 expression was associated with HER2 negativity (*p* = 0.036) and ER positivity (*p* = 0.032).

Nuclear, but not cytoplasmic, Keap1 expression was increased during the NACT (*p* = 0.022). Pre-NACT nuclear Keap1 expression was also connected with better tumor differentiation (*p* = 0.029). Higher nuclear Keap1 expression in postoperative samples was associated with the presence of bilateral breast cancer (*p* = 0.026). Furthermore, expressions of Keap1 and Trx were strongly connected with each other in postoperative cytoplasmic (*p* = 0.00048; *r* = 0.504) and nuclear (*p* = 0.0024; *r* = 0.446) staining. Pre-NACT and postoperative nuclear Keap1 H scores also showed a positive correlation (*p* = 0.011; *r* = 0.376).

Nuclear Prx I expression was highly increased during NACT (*p* = 0.00028), with all samples postoperatively showing some Prx I expression. The nuclear staining of Prx I in core needle biopsies correlated inversely with the size of a resected tumor (*p* = 0.00052; *r* = −0.550). Also, cytoplasmic staining of the Prx I in resected tumor samples had an inverse correlation with the amount of nodal metastasis (*p* = 0.0024; *r* = −0.472).

We also examined whether the change in antibody H scores between core needle biopsy and postoperative expression was associated with clinicopathological parameters. Increased cytoplasmic Trx expression was associated with larger primary tumor size preoperatively (*p* = 0.037; *r* = 0.396) and postoperatively (*p* = 0.029; *r* = 0.389).

### 3.1. Survival Analysis

In ROC analysis, the optimal cut-off H score of 15.0 was defined for pre-NACT nuclear Trx expression with regard to DFS. Likewise, ROC analysis confirmed an optimal cut-off H score of 22.5 for pre-NACT nuclear Keap1 in terms of DFS.

**Figure 2:**
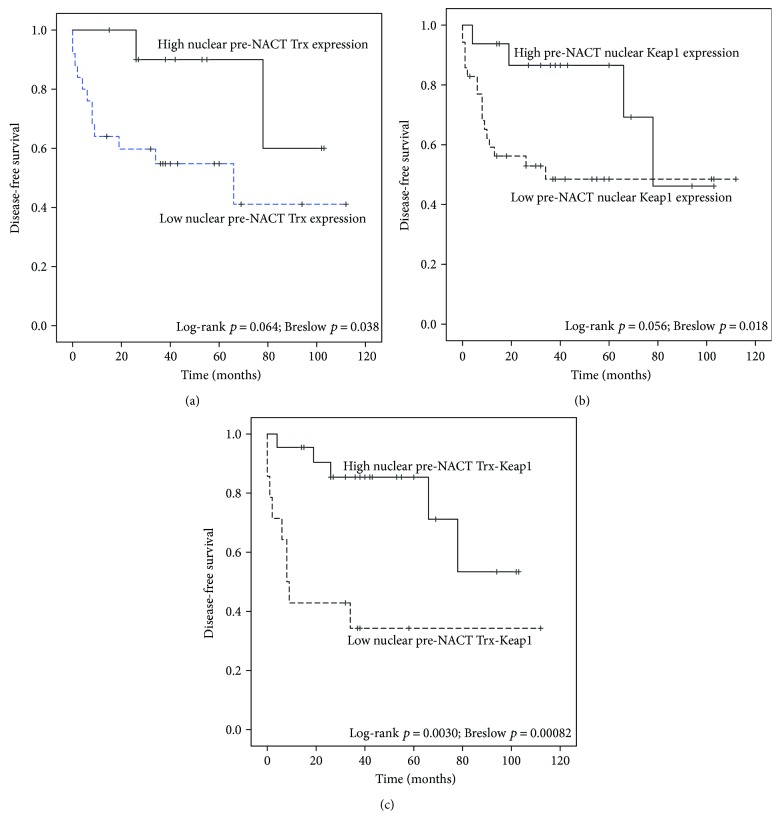
Kaplan-Meier curves comparing preneoadjuvant chemotherapy (NACT) expression of Trx (a) and Keap1 (b). Cut-offs for low and high expression have been generated with ROC analysis. In part (c), cases with both low nuclear pre-NACT Keap1 and low nuclear pre-NACT Keap1 have been set against other patients. Crosses indicate censored cases.

Higher pre-NACT nuclear Trx and nuclear Keap1 expressions predicted better DFS (log-rank *p* = 0.064; Breslow *p* = 0.038 and log-rank *p* = 0.056; Breslow *p* = 0.018, resp.) (Figure 2). When pre-NACT nuclear Trx expression and pre-NACT nuclear Keap1 expression were combined as a single factor (0 = low expression for both, 1 = high expression for Trx and/or Keap1), low expression of both Trx and Keap1 predicted poor DFS highly significantly (log-rank *p* = 0.0030; Breslow *p* = 0.00082). No significant associations between the studied markers and BCSS were found.

## 4. Discussion

Predictive factors for breast cancer chemotherapy are scarce, to date including mainly immunohistochemical surrogates, such as ER negativity and high Ki-67 for the identification of more chemosensitive luminal B-type breast cancer [23]. Our goal was to determine if the main regulators of the cellular redox state would have an impact on neoadjuvant chemotherapy effectiveness or patient outcome in the patients with locally advanced, primarily inoperable breast cancer.

The Trx system, including Trx, thioredoxin reductase (TrxR), and thioredoxin-interacting protein (TxNIP), not only participates to the early phases of breast carcinogenesis but is also connected to ER negativity, high proliferation, and poor survival in breast cancer and is involved in chemotherapy resistance *in vitro* [24, 25]. In lymphomas, siRNA targeted against Trx led to the sensitization of the tumor to doxorubicin which resulted in cell growth inhibition while in stomach cancer, Trx expression has been linked to multidrug resistance [26, 27]. Woolston et al. previously assessed the predictive and prognostic value of Trx family proteins in anthracycline-based NACT-treated breast cancer patients. Although predictive markers were not recognized, patients with high immunohistochemically determined TxNIP or TrxR expression had dismal outcomes [28].

In our patients, both nuclear and cytoplasmic Trx expressions increased in tumor tissue during the NACT. Increased cytoplasmic Trx expression during NACT also associated with a larger primary tumor size. This may reflect Trx-mediated chemoresistance during the therapy; alternatively, Trx induction may contribute to the increased proliferation and apoptosis resistance in various cancers [12]. Furthermore, pre-NACT nuclear Trx expression was associated with the prolonged DFS, but only with borderline significance. Trx enhances anthracycline-mediated apoptosis in breast cancer MCF-7 cells, and, again, TrxR predicts better distant metastasis-free survival in clinical breast cancer material [28, 29], although the prognostic role of Trx has been less clear.

Keap1 is a cytosolic or nuclear cysteine-rich protein, which in unstressed conditions targets newly synthesized Nrf2 to proteosomal degradation [30]. Under oxidative stress, several Cys residues in Keap1 are oxidized; consequently, Nrf2 becomes stable and bypasses degradation which ultimately results in widespread expression of antioxidant proteins [31]. Keap1 somatic mutations have been linked to chemoresistance in various carcinomas, although Keap1 protein expression in this context is less studied [32–35]. Higher nuclear Keap1 expression in pre-NACT samples predicted prolonged DFS in our patients, suggesting the block of excess Nrf2 function and the suppression of subsequent antioxidant induction. Rather surprisingly, no nuclear Nrf2 expression was noted in the current study, which may at least partially be linked with technical reasons. Analogous observations of Keap1 protein overexpression and better survival have been reported from pancreatic cancer and from squamous non-small-cell lung carcinoma [36, 37].

In the context of breast cancer, aberrant Keap1 methylation was found as an independent prognostic factor of better DFS (HR = 0.082), specifically in the patients treated with anthracycline/taxane-based chemotherapy [38]. In the same paper, Keap1 methylation associated with an increased risk of BCSS in the subset of patients with triple-negative breast cancer. We have earlier reported that stronger immunohistochemical Keap1 expression is a poor prognostic factor for BCSS [39]. The current results therefore differ from our earlier observations. However, in the patients comprising this study, the prognostic value of Keap1 expression was noted for untreated preoperative samples, which, according to our knowledge, has not been explored previously. Notably, the number of samples showing nuclear Keap1 positivity nearly doubled during NACT, which suggests a role for Keap1 as a gatekeeper for Nrf2 as a response to cytotoxic therapy. The same phenomenon of markedly elevated Keap1 levels during the NACT was recently detected in ovarian cancer patients [40].

There was a strong positive correlation between Keap1 and Trx in our study, which supports the recent hypothesis that Trx as highly redox-reactive protein also maintains the active state of cysteine-rich Keap1 [41]. Patients with both low Trx and Keap1 expressions had a dismal prognosis in our current patients, with 53% having either distant or local relapse during the first nine months after diagnosis. Although there were no progressive diseases during NACT in our study cohort, the high proportion of early relapses in low-Keap1/Trx patients suggests a negative impact for the simultaneous loss of Trx and Keap1 due to chemosensitivity. Nevertheless, the low number of patients with both Keap1 and Trx core needle biopsy immunostainings available (*n* = 36) limits the power of the analysis making multivariate analysis unreliable. Another potential limitation of the study may be that due to limited sample size, we were unable to assess biological subgroups separately.

The role of Prx I in cancer development is considerably studied but to date not yet fully defined. Knockdown/knockout mice with Prx I deficiency are prone to elevated ROS amounts and development of cancer [42]. Thus, high Prx levels may protect DNA against mutations and carcinogenesis. On the other hand, Prx I gene expression induction in MCF-7 cells has been linked to the platinum resistance, which is likely due to the elimination of chemotherapy-induced ROS [43]. Furthermore, Prx I knockdown in HeLa cells induces the efficacy of beta-lapachone, an ROS-generating experimental chemotherapeutic agent [44]. In clinical samples, Prx I appears to associate with worse prognosis in pancreatic cancer, cholangiocarcinoma, hepatocellular carcinoma, and early-stage non-small-cell lung cancer [45–48]. In contrast to this, Prx I was an independent predictor of improved outcomes in a large set of ER-positive breast cancers [49]. Breast cancer-suppressive properties of Prx I have been proposed to be mediated via the inhibition of c-Myc activation and p53-dependent cytotoxicity [9, 10]. Supporting this cancer-specific role of Prx I, we observed that elevated Prx I expression was associated with both smaller primary tumor size and lower number of lymph node metastases. Additionally, we noted a significant induction of Prx I during NACT, which may reflect cancer cell adaptation to oxidative conditions [50, 51]. This would be in line with the previous mouse model data showing that doxorubicin increases the mRNA and protein expressions of Prx I, II, III, V, and VI through metallothionein activation [52].

## 5. Conclusions

There appears to be significant antioxidant enzyme upregulation during breast cancer NACT. Due to the restricted sample size, the current study is mainly hypothesis generating and applies only to patients with primary inoperable breast cancer. If confirmed in larger and preferably in prospective settings, especially Keap1 and Trx expression in chemotherapy-naïve patients may serve as predictive or prognostic biomarkers.

## Figures and Tables

**Table 1:** Patient characteristics.

	Number of patients	Percentage or range
*Mean years of age at diagnosis*	56.4	32–77
*Menopausal status*	**53**	**100**
Premenopausal	17	32.1
Postmenopausal	28	52.8
Not known	8	15.1
*Bilateral breast cancer*	**53**	**100**
Bilateral breast cancer	4	7.5
Unilateral breast cancer	49	92.5
*NACT received*	**53**	**100**
Docetaxel + doxorubicin	20	37.7
Docetaxel + trastuzumab	14	26.4
Docetaxel + epirubicin	3	5.7
Other chemotherapy	16	30.2
*Median number of neoadjuvant cycles*	6.0	2–16
*Surgical procedure*	**53**	**100**
Mastectomy and axillary evacuation	53	100
*Adjuvant chemotherapy*	**53**	**100**
Cyclophosphamide + epirubicin + fluorouracil	11	20.8
Other chemotherapy	18	34
No adjuvant chemotherapy	24	45.3
*Radiotherapy*	**53**	**100**
Yes	52	98.1
No	1	1.9
*Adjuvant endocrine therapy*	**53**	**100**
Tamoxifen	10	18.9
Aromatase inhibitor	23	43.4
GnRH analogue + tamoxifen	5	9.4
GnRH analogue + aromatase inhibitor	1	1.9
Tamoxifen and aromatase inhibitor (sequentially)	2	3.8
No adjuvant hormonal therapy	12	22.6
*Recurrence status*	**53**	**100**
Distant	20	37.7
Local	3	5.6
No recurrence	30	56.6

**Table 2:** Pre- and postoperative tumor sizes.

	Mean size (mm) and number of patients	Range
Tumor size at the time of diagnosis	54.6 (50)	10–140 mm
Preoperative tumor size	31.2 (47)	0–90 mm
Postoperative tumor size	39.1 (52)	0–150 mm

**Table 3:** Tumor properties. Estrogen receptor (ER), progesterone receptor (PR), Ki-67, and HER2 expressions and grade are reported as in postoperative PAD. If unavailable in postoperative PPS, the assessment from the core needle biopsy is reported.

	Number of patients	Percentage or range
*Response to NACT*	**52** ^∗^	**100**
Complete response	6	11.3
Partial response	44	83.0
Stable disease	2	3.8
Progression	0	0
*Histopathological grade*	**53**	**100**
Grade 1	1	1.9
Grade 2	19	35.8
Grade 3	19	35.8
No information or no viable cancer cells	14	26.4
*ER status*	**53**	**100**
Negative (0%)	9	17.0
Weak (1–9%)	8	15.1
Moderate (10–59%)	2	3.8
High (>59%)	34	64.2
*PR status*	**53**	**100**
Negative (0%)	23	43.4
Weak (1–9%)	5	9.4
Moderate (10–59%)	3	5.6
High (>59%)	22	41.5
*Ki-67 status*	**53**	**100**
Negative (<5%)	6	11.3
Weak (5–14%)	12	22.6
Moderate (15–30%)	12	22.6
High positive (>30%)	22	41.5
No information or no viable cancer cells	1	1.9
*HER2 status*	**53**	**100**
Negative	37	69.8
Positive (confirmed with CISH)	16	30.2
*Median number of metastatic lymph nodes*	**2.0**	**0–20**
*Distant metastases at the time of diagnosis*	**53**	**100**
Absent	43	81.1
Present	10	18.9

^∗^The information of pre-NACT tumor size was missing from one patient.

**Table 4:** Antigen staining in different cell compartments. The percentages represent cases showing any immunopositivity.

Target protein	Cytoplasmic staining, pretreatment (%)	Cytoplasmic staining, posttreatment (%)	Nuclear staining, pretreatment (%)	Nuclear staining, posttreatment (%)
Keap1	100.0	100.0	45.3	88.9
Nrf2	94.6	100.0	0	0
Prx I	100.0	100.0	45.9	100.0
Trx	94.6	100.0	64.9	81.8
